# Regulation of p53 Level by UBE4B in Breast Cancer

**DOI:** 10.1371/journal.pone.0090154

**Published:** 2014-02-26

**Authors:** Ying Zhang, Yanrong Lv, Yongyang Zhang, Haidong Gao

**Affiliations:** 1 Department of Breast Surgery, QiLu Hospital, Jinan, China; 2 Emergency Department, Shandong Jining No.1 People's Hospital, Jining, China; German Cancer Research Center, Germany

## Abstract

p53 is possibly the most important mammalian tumor suppressor and it is mutated or lost in more than half of all human cancers. The stability of p53 is primarily determined by the RING domain E3 ubiquitin ligase Hdm2, which targets p53 for proteasomal degradation, restraining the potent activity of p53 and enabling cell survival and proliferation. UBE4B has been shown to physically interact with p53 and Hdm2 and to negatively regulate p53 stability and function. However, no one has determined whether UBE4B promotes p53 degradation in breast cancer. In this study, UBE4B promoted the degradation and ubiquitination of p53 to inhibit the apoptosis of cancer cells and promote tumorigenesis. Our results indicate that UBE4B regulates p53 in breast cancer and could be a viable target for developing new therapeutic strategies for breast cancer treatment.

## Introduction

Ubiquitin (Ub) is a highly conserved 8 kDa protein that covalently attaches to the lysine (Lys) residues of target proteins. Ubiquitylation is performed by Ub-activating enzymes (E1s), Ub conjugating enzymes (E2s), and Ub ligases (E3s) [1], [4], [5]. Proteins are reliant on the quantity of attached Ub moieties and can be modified through mono-ubiquitination or polyubiquitination. The former means that a single Ub moiety is attached to one or several lysine residues on the substrate; whereas the latter means that the substrate protein is modified by a chain of over four Ub moieties linked to one of the seven lysine residues in Ub [2]. The activity and degradation of proteins that control key processes, such as cell cycle progression, differentiation, and apoptosis, are regulated by the Ub/proteasome system [3]. The Ub chain assembly factor (E4), a new class of ubiquitylation enzyme, has recently been discovered and is shown to be required in the degradation of certain types of substrate through a Ub fusion degradation pathway, designated as UFD [4], [5]. The UBE4B gene, which is located in the 1p36 region and encodes an E3/E4 ubiquitin ligase, was previously known as UFD2 in yeast [6]. This gene catalyzes Ub chain assembly in conjunction with E1, E2, and E3 and binds to the Ub moieties of preformed conjugates [5].

The loss of p53 has a vital role in carcinogenesis, and the regulation of p53 expression and its stability are essential to maintain normal cell growth [7]. Hdm2 is a critical negative regulator of p53 and only catalyzes p53 monoubiquitination or multiple monoubiquitination. Hdm2 does not polyubiquitinate p53 [7]–[8]. In 2011, Wu et al. showed that UBE4B physically interacts with p53 and Hdm2 and negatively regulates p53 stability and function [7]. However, no one has reported on whether UBE4B promotes p53 degradation in breast cancers. The results of these studies suggest that UBE4B-mediated p53 degradation may contribute to the poor prognosis of breast cancer patients and that UBE4B expression may be a marker the response of breast cancer to treatment.

## Materials and Methods

### Cell and tumor specimens

Two human breast cancer cell lines, namely, MCF-7 and SK-BR-3, were cultured at 37°C in Dulbecco's modified Eagle's medium (DMEM) supplemented with 10% FBS(Hyclone, USA) under a 5% CO2 atmosphere. Breast cancer tissue samples were obtained from patients who underwent surgical resection at the Department of Breast Surgery at the QiLu Hospital of Shandong University. This study was approved by the Human Ethics Review Committee of the QiLu Hospital of Shandong University. All participants provided their written informed consent to participate in this study.

### Plasmids and transfection

The UBE4B and Hdm2 expression plasmids were purchased from Sino Biological Inc (China). The siRNAs (UBE4B: 5′-GCAACTAGACACCGCGAAA-3′ and Hdm2:5′-GACAAAGAAGAGAGTGTGG-3′) were purchased from GenePharm (China). For transient transfection, 1.0×10^5^ cells were plated into 12-well plates and cultured for 24 h in DMEM supplemented with 10% FBS before transfection. The cells were then transfected with the expressing plasmid using TurboFect in vitro transfection reagent (Fermentas, CA), and the siRNA were transfected using Lipofectamine 2000 (Invitrogen, USA) according to the manufacturer's instructions.

### Western blot analysis

The proteins were separated using sodium dodecyl sulfate polyacrylamide gel electrophoresis (SDS–PAGE) and transferred onto a polyvinylidene fluoride membrane. The membranes were blocked in 5% non-fat milk and incubated overnight with primary antibodies. The membranes were then incubated for 1 h with the HRP-conjugated secondary antibodies at room temperature and shaken slightly. Immunoreactive bands were visualized using SuperSignal West Pico Chemiluminescent Substrates (Thermo, USA).

### Immunoprecipitation

The cultured cells were lysed in 4-(2-hydroxyethyl)-1-piperazineethanesulfonic acid (HEPES) buffer composed of 50 mM HEPES (pH 7.2), 250 mM NaCl, 10% glycerol, 1% NP-40, 1.0 mM ethylenediaminetetraacetic acid, 0.5 mM dithiothreitol, and 10 mM phenylmethylsulfonyl fluoride supplemented with Protease Inhibitor Cocktail Tablets (Roche). The cell lysates were incubated for 8 h at 4°C on agarose with the indicated primary antibodies and then washed according to the manufacturer's protocol. The samples were separated using SDS-PAGE and western blot analysis.

### *In vivo* ubiquitylation assay

The cells were treated with 20 μM MG132 proteasome inhibitor for 6 h. The cells were washed with PBS and lysed in 0.5 ml of HEPES buffer (20 mM HEPES, pH 7.2, 50 mM NaCl, 1 mM NaF, 0.5% Triton-X 100) supplemented with 0.1% SDS and protease-inhibitor cocktail (Roche, Germany). The lysates were centrifuged to obtain the cytosolic proteins. Briefly, individual samples were incubated for 6 h with primary antibodies and then with protein A/G-agarose beads (Santa Cruz) for another 8 h at 4°C. The beads were then washed three times with HEPES buffer. The proteins were released from the beads by boiling in a 40 μl of 2× SDS-PAGE sample buffer for 10 min. The samples were subjected to western blot analysis.

### Apoptosis assays

MCF-7 cells were cotransfected with a p53 expression plasmid combined with an empty vector, UBE4B, and Hdm2 for 48 h. Another group of MCF-7 cells were transfected with UBE4B siRNA, Hdm2 siRNA, or control siRNA. The cells were then treated with cisplatin (10 μM, 24 h, Sigma–Aldrich, USA). After incubation, the cells were washed with PBS solution and stained in the dark with Annexin V and propidium iodide (PI) for 10 min at room temperature. Apoptotic cells were then determined via flow cytometry.

### Nude mouse experiments

A total of 20 female 6-week-old nude mice weighing 20 g to 22 g were purchased from the animal center of Shandong University. The mice were fed a standard rodent diet and provided with water in an aseptic laminar flow room at 25°C. Then, 100 µl of the cell suspensions (containing 1×10^7^logarithmic growth phase tumor cells) were subcutaneously injected into the mice. Tumor growth was observed every week for 6 weeks by measuring the length and width using a Vernier caliper. Tumor volume was calculated from the lengths and widths using the following formula: tumor volume  =  (length × width^2^)/2. The Animal Ethics Review Committee of Shandong University approved the animal research of this study.

### Statistical analysis

Data are presented as mean ± standard deviation. A t-test was used to analyze all results. For all tests, differences with *P*<0.05 were considered statistically significant.

## Results

### UBE4B and p53 are expressed in breast cancer tissues and UBE4B enhances p53 ubiquitination

**Figure 1:**
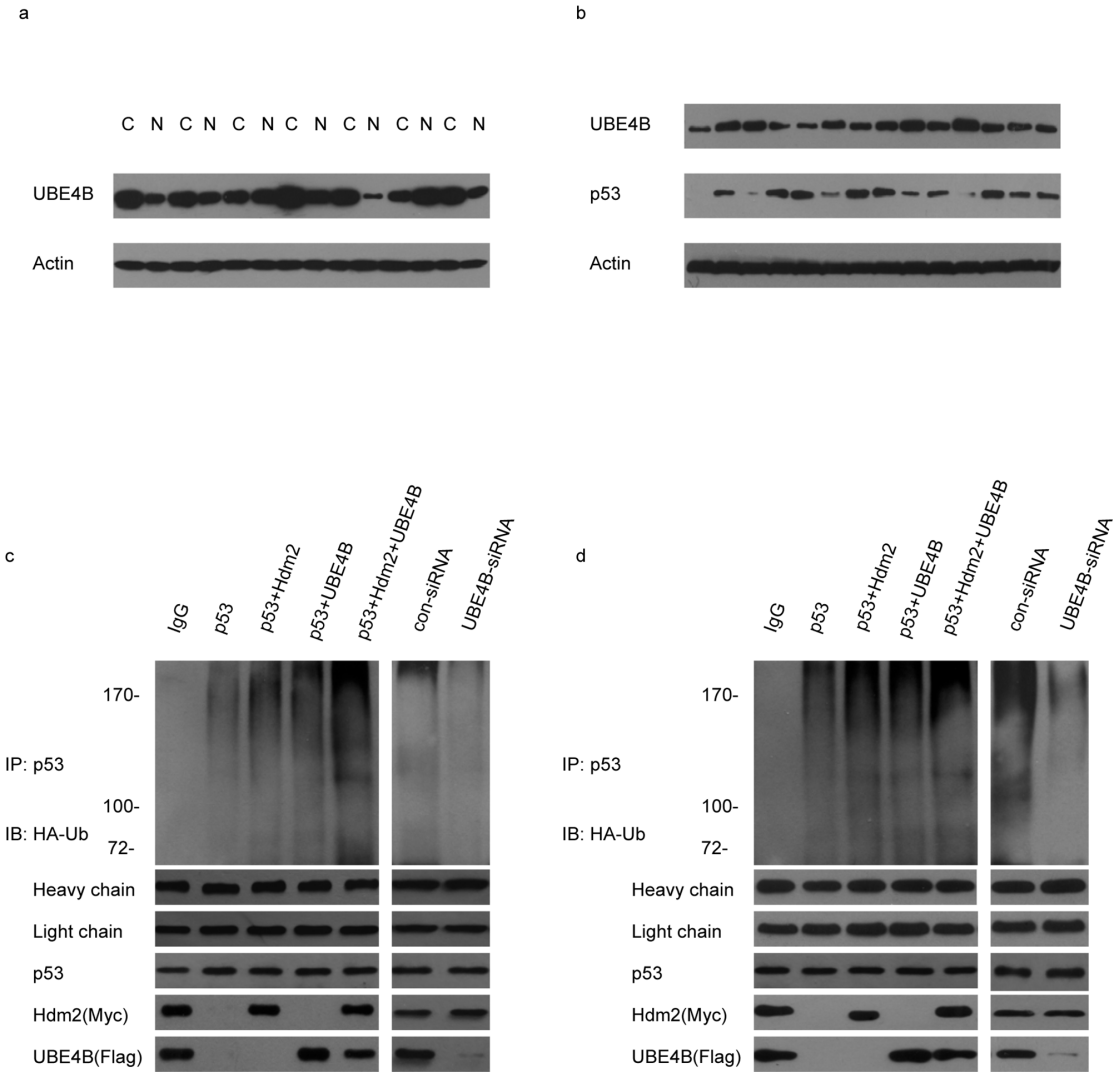
The expression of UBE4B and p53 in breast cancer tissues and UBE4B enhances p53 ubiquitination. (a) Western blot analysis using UBE4B-specific antibodies showing UBE4B protein expression in normal breast tissues and in breast cancer tissues (C: breast cancer tissue; N: normal breast tissue). (b) Western blot analysis of breast cancer tissues with UBE4B-specific and p53-specific antibodies. (c) MCF-7 cells were cotransfected with HA-Ub, p53, Hdm2, and UBE4B. p53 was immunoprecipitated and analyzed by Western blot with a HA-specific antibody. (d) The same experiment was repeated using SK-BR-3 cells.

We detected UBE4B protein expression in normal breast tissues and breast cancer tissues. UBE4B expression was higher in breast cancer tissues than in normal breast tissues (Fig. 1a). We then examined the protein expression of UBE4B and p53 in the breast cancer tissues using western blot analysis. The levels of p53 and UBE4B mainly showed an opposite state (Fig. 1b). HA-tagged ubiquitin (HA-Ub) was coexpressed in MCF-7 cells with plasmids encoding p53 or in combination with either UBE4B, Hdm2, or UBE4B and Hdm2. p53 was immunoprecipitated and analyzed via western blot analysis using anti-HA antibodies to detect ubiquitinated p53. The p53 protein was heavily ubiquitinated in the presence of Hdm2 or UBE4B and was more heavily ubiquitinated by the coexpression of Hdm2 and UBE4B. The level of p53 ubiquitination was evidently decreased when p53 and UBE4B-siRNA were coexpressed (Fig. 1c). The same condition was observed in the SK-BR-3 cells (Fig. 1d).

### Interactions of UBE4B with Hdm2 and with p53 *in vivo*

**Figure 2:**
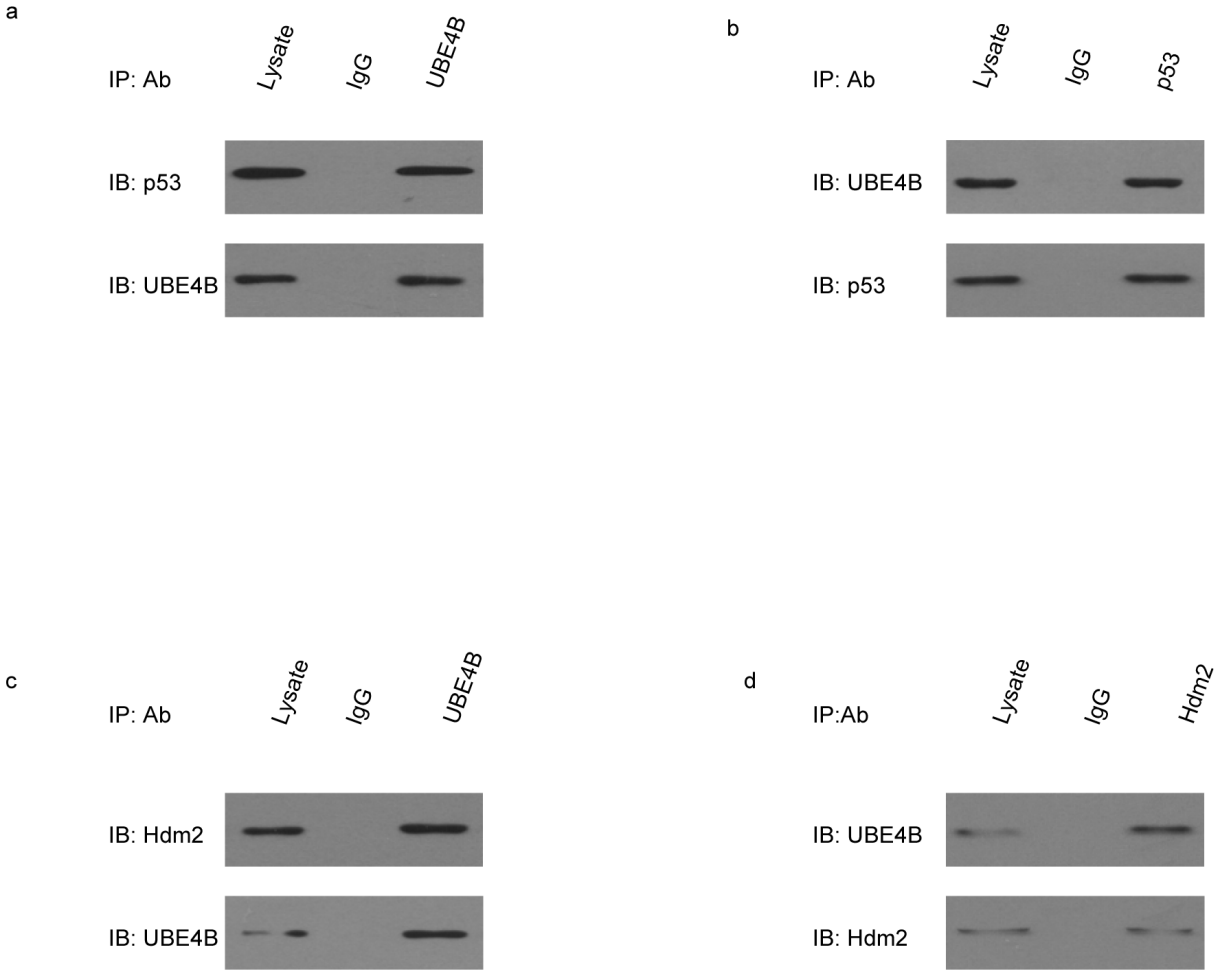
Interactions of UBE4B with Hdm2 and p53 *in vivo*. (a) Western blot analysis using UBE4B-specific and p53-specific antibodies after p53 coimmunoprecipitation of from MCF-7 cells and lysates using UBE4B-specific or IgG-specific antibodies. (b) Western blot analysis using anti-UBE4B or anti-p53 antibodies after UBE4B coimmunoprecipitation from MCF-7 cells and lysates using anti-p53 or anti-IgG antibodies. (c) Western blot analysis using UBE4B-specific or Hdm2-specific antibodies after Hdm2 coimmunoprecipitation from MCF-7 cells and lysates using UBE4B-specific or IgG-specific antibodies. (d) Western blot using anti-UBE4B or anti-Hdm2 antibodies after UBE4B coimmunoprecipitation from MCF-7 cells and lysates using anti-Hdm2 or anti-IgG antibodies.

The interaction of UBE4B with Hdm2 and with p53 was tested in MCF7 cells. Immunoprecipitation was performed on the cultured MCF7 cells using anti-UBE4B antibodies. The physiologic interaction between UBE4B and p53 was evaluated using western blot analysis with the respective antibodies (Fig. 2a). Immunoprecipitation in MCF7 cells was conducted using the anti-p53 antibody (Fig. 2b). The results showed that UBE4B interacts with p53. Similar experiments were conducted to determine whether UBE4B interacts with Hdm2 (Figs. 2c and 2d). The results validated the hypothesis that UBE4B interacts with Hdm2. The combined results proved that UBE4B interacts with both p53 and Hdm2.

### UBE4B negatively regulates p53 and promotes p53 degradation in the presence of Hdm2

**Figure 3:**
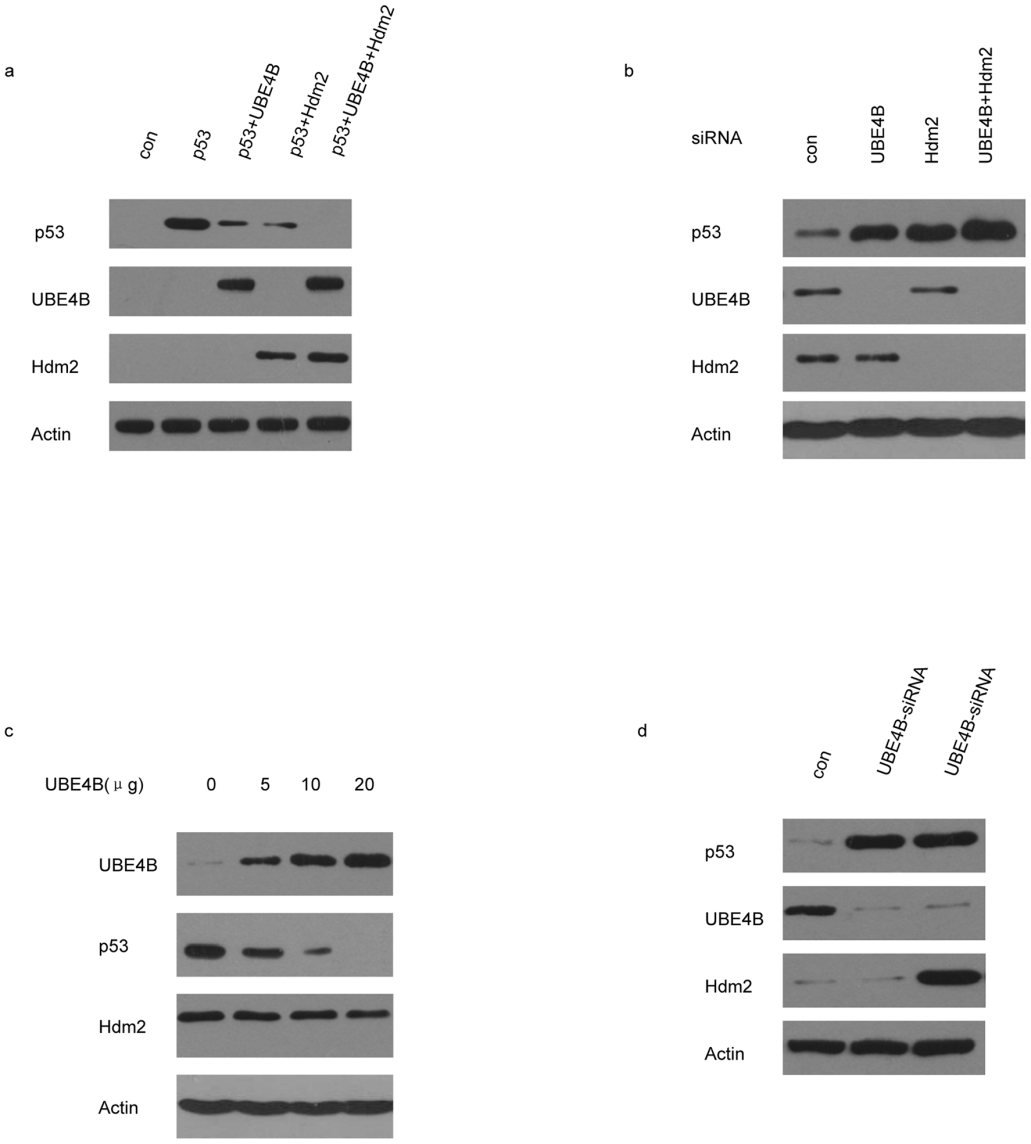
UBE4B negatively regulates p53 and promotes p53 degradation in the presence of Hdm2. (a) Plasmids expressing Flag-UBE4B, Myc-Hdm2 with p53 were cotransfected into p53^−/−^ MCF-7 cells (p53 KO), and analyzed via western blot analysis using Flag-specific, Myc-specific, and p53-specific antibodies. (b) MCF-7 cells were cotransfected with UBE4B-siRNA or Hdm2-siRNA, and then analyzed via western blot analysis using p53-specific, UBE4B-specific, or Hdm2-specific antibodies. (c) The increasing amounts of UBE4B plasmid were transfected into WT p53-expressing MCF-7 cells. The transfected cells were analyzed via western blot analysis using anti-p53, anti-UBE4B and anti-Hdm2 antibodies, with actin as the internal reference gene. (d) MCF-7 cells were transfected with control siRNA or UBE4B siRNA. After 30 h, the cells were transfected further with Hdm2 expression plasmid and analyzed via western blot analysis using p53-specific, UBE4B-specific, and Hdm2-specific antibodies.

p53^−/−^ MCF-7 cells (p53 KO) were cotransfected with Flag-UBE4B or with Myc-Hdm2 and p53. The amount of p53 protein was reduced by UBE4B and Hdm2 and was more reduced when UBE4B and Hdm2 were coexpressed (Fig. 3a). We transfected MCF-7 cells with UBE4B siRNA or Hdm2 siRNA to determine whether UBE4B is required for regulating the *in vivo* p53 protein levels. The expected results were as predicted (Fig. 3b). Increasing amounts of UBE4B were transfected into WT p53-expressing MCF-7 cells, which showed that UBE4B-induced p53 degradation is dose dependent (Fig. 3c). We transfected MCF-7 with UBE4B siRNA or the control siRNA to determine whether UBE4B is crucial for Hdm2-mediated p53 degradation in breast cancer cells. These cells were transfected further with the Hdm2 expression plasmid after 30 h. The results suggest that Hdm2-induced p53 degradation in breast cancer cells requires UBE4B (Fig. 3d).

### UBE4B inhibits p53-dependent apoptosis and promotes tumorigenesis

**Figure 4:**
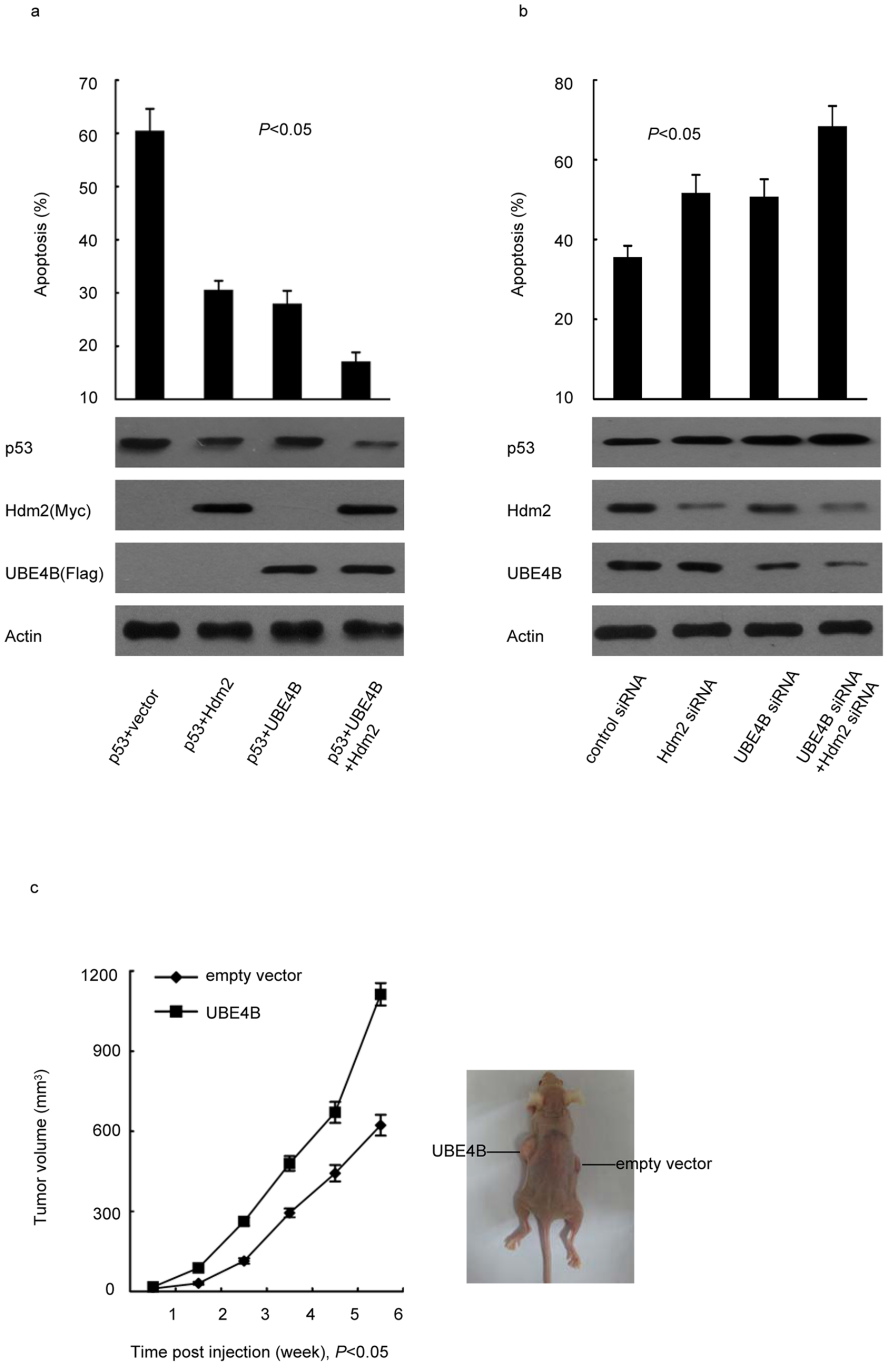
UBE4B inhibits p53-dependent apoptosis and promotes tumorigenesis. (a) p53^−/−^ MCF-7 cells (p53 KO) were cotransfected with a p53 expression plasmid in combination with an empty vector, Flag-UBE4B, and Myc-Hdm2 (* *P*<0.05). (b) MCF-7 cells were transfected with UBE4B siRNA, Hdm2 siRNA, or control siRNA. (a and b) The cells were treated with cisplatin after 48 h, and all of cells were then analyzed via flow cytometry (* *P*<0.05). (c) Six-week-old female nude mice weighing 20 g to 22 g were subcutaneously injected with 1×10^7^ MCF-7 cells expressing vector encoding UBE4B or an empty vector. Tumor growth was observed every week for 6 weeks by measuring their length and width using a Vernier caliper. Tumor volume  =  length × width^2^/2 (*P*<0.05).

The decrease in breast cancer cells apoptosis induced by UBE4B overexpression in contrast to that of p53 indicated that UBE4B inhibits cancer cell apoptosis (Fig. 4a). Coexpression of UBE4B and Hdm2 more significantly prevented apoptosis than cotransfected with UBE4B-siRNA, Hdm2-siRNA, or UBE4B siRNA and Hdm2 siRNA (Fig. 4b). To test the *in vivo* tumorigenic efficacy of UBE4B, we established a xenograft animal model in nude mice. We subcutaneously injected MCF-7 expressing vector encoding UBE4B or an empty vector into the mice. We found that UBE4B-overexpressing tumors have significantly faster growth rates than tumors with normal UBE4B expression. By contrast, the tumors formed by cells transfected with empty vector were much smaller (Fig. 4c).

## Discussion

The p53 protein and its gene, *TP53*, were identified in 1979 [9]–[11]. p53 plays a critical role in cancer prevention because it suppresses tumorigenesis by inducing cell cycle arrest and apoptosis through its transcriptional activity. p53 is a tumor suppressor genes most frequently inactivated in cancer [12]. Aside from its role in tumorigenesis, p53 also affects the effectiveness of platinum therapy [13]. Breast cancer is the most common cancer in women worldwide [14]. Breast cancer is over 100 times more common in women than in men. The efficacy of p53 activity represents a vulnerable link for preventing tumorigenesis in the breast epithelium [15]. Breast cancer cells with wild-type p53 often have high levels of the oncogenic protein Hdm2, which suggests that Hdm2 may block p53 function [16]–[18]. The Hdm2 oncoprotein is a well-studied E3 Ub that targets the p53 tumor suppressor protein for proteasomal degradation [19]. The ubiquitination of p53 by Hdm2 generally inhibits p53 function. Hdm2 only catalyzes p53 monoubiquitination or multiple monoubiquitination *in vitro* and *in vivo*. Hdm2 does not polyubiquitinate p53 *in vitro* [20], [21]. In 2011, Wu et al. reported that UBE4B is essential for Hdm2-mediated p53 polyubiquitination and degradation [7], which suggests that increased UBE4B activity may be oncogenic by reducing the abundance of p53. In this study, we revealed the physiological significance of UBE4B in p53 degradation in breast cancer.

The UBE4B gene is located in the 1p36 region and encodes an E3/E4 Ub ligase [6] that promotes the degradation of a pathologic form of ataxin-3 [22]. UBE4B levels reportedly influenced neuroblastoma tumor cell proliferation, and UBE4B was overexpressed in various brain tumors [7], [23]. In this paper, we first reported UBE4B protein expression in breast cancer tissues and we determined that breast cancer tissues have significantly higher levels of UBE4B than normal tissues. The amount of p53 was inversely proportional to that of UBE4B. Furthermore, the experiments determined that UBE4B ubiquitinates p53 and promotes the Hdm2-mediated ubiquitination of p53 in the breast cancer cell line. In addition, UBE4B physically interacted with Hdm2 and p53 *in vivo*, and UBE4B negatively regulates p53. Finally, UBE4B inhibits p53-dependent apoptosis and promotes tumorigenesis in breast cancer. All of the results illustrate that UBE4B is essential for Hdm2-induced p53 degradation and UBE4B may promote the formation of breast cancer.

Overall, we have identified how UBE4B can reduce the amount of p53 protein and promote the Hdm2 mediated ubiquitination of p53 in breast cancer. These findings proved further that UBE4B is an oncogene and may promote the formation of breast cancer tumors. UBE4B will ultimately provide the most effective way to take advantage of the ever-increasing list of new biological therapies for breast cancer.
