# Correction: Regulation of p53 Level by UBE4B in Breast Cancer

**DOI:** 10.1371/journal.pone.0099968

**Published:** 2014-06-04

**Authors:** The PLOS ONE Staff

There is an error in the affiliation for authors Ying Zhang, Yanrong Lv, and Haidong Gao. The first affiliation should be listed as the Department of Breast Surgery, QiLu Hospital of Shandong University, Jinan, China. The updated list can be viewed below:

Ying Zhang^1^, Yanrong Lv^1^, Yongyang Zhang^2^, Haidong Gao^1^*

1 Department of Breast Surgery, QiLu Hospital of Shandong University, Jinan, China

2 Emergency Department, Shandong Jining No.1 People’s Hospital, Jining, China

* E-mail: haidongao@163.com
