# Correction: Regulation of p53 Level by UBE4B in Breast Cancer

**DOI:** 10.1371/journal.pone.0125136

**Published:** 2015-04-13

**Authors:** The PLOS ONE Staff

The first two authors, Ying Zhang and Yanrong Lv, should be noted as contributing equally to this work. The publisher apologizes for the error.
